# Features of Copper and Gold Nanoparticle Translocation in *Petroselinum crispum* Segments

**DOI:** 10.3390/nano13111754

**Published:** 2023-05-28

**Authors:** Alexandra Peshkova, Inga Zinicovscaia, Liliana Cepoi, Ludmila Rudi, Tatiana Chiriac, Nikita Yushin, Alexander Sohatsky

**Affiliations:** 1 Joint Institute for Nuclear Research, 6 Joliot-Curie Str., 141980 Dubna, Russia; peshkova.alexandra92@gmail.com (A.P.); ynik_62@mail.ru (N.Y.); sohatsky@jinr.ru (A.S.); 2 Doctoral School Biological, Geonomic, Chemical and Technological Science, State University of Moldova, 60 Alexei Mateevici Str., MD-2009 Chisinau, Moldova; 3 Horia Hulubei National Institute for R&D in Physics and Nuclear Engineering, 30 Reactorului Str., 077125 Măgurele, Ilfov, Romania; 4 Institute of Chemistry, 3 Academiei Str., MD-2028 Chisinau, Moldova; 5 Institute of Microbiology and Biotechnology, Technical University of Moldova, 1 Academiei Str., MD-2028 Chisinau, Moldova; liliana.cepoi@imb.utm.md (L.C.); ludmila.rudi@imb.utm.md (L.R.); tatiana.chiriac@imb.utm.md (T.C.)

**Keywords:** copper nanoparticles, gold nanoparticles, uptake, parsley, biochemistry

## Abstract

The application of metal nanoparticles in industry and medicine results in their release into the environment, which can have a negative impact on human health. The effects of gold (AuNPs) and copper (CuNPs) nanoparticles at the concentration range of 1–200 mg/L on parsley (*Petroselinum crispum*) under conditions of root exposure and their translocation in roots and leaves were investigated in a 10-day experiment. The content of copper and gold in soil and plant segments was determined using ICP-OES and ICP-MS techniques, while the morphology of nanoparticles was analyzed using transmission electron microscopy. Differences in the nanoparticle uptake and translocation were observed: CuNPs mainly accumulated in soil (4.4–465 mg/kg), while accumulation in the leaves were at the control level. AuNPs mainly accumulated in soil (0.04–108 mg/kg), followed by roots (0.05–45 mg/kg) and leaves (0.16–53 mg/kg). The influence of AuNPs and CuNPs on the biochemical parameters of parsley was on the content of carotenoids, the levels of chlorophyll, and antioxidant activity. Application of CuNPs even at the lowest concentration led to a significant reduction in carotenoids and total chlorophyll content. AuNPs at low concentrations promoted an increase in the content of carotenoids; however, they also significantly reduced it at concentrations higher than 10 mg/L. To our knowledge, this is the first study of the effect of metal nanoparticles on parsley.

## 1. Introduction

The relevance of the study of the influence of metal nanoparticles (NPs) on various biological objects and human health is explained by their active application in various fields of industry and medicine due to their unique electrical, optical, and mechanical properties [1,2,3,4,5,6]. For example, AuNPs play an important role in cancer treatment and diagnosis, because they are used as contrast agents for tumor visualization, as radiation therapy amplifiers, and in the targeted delivery of drugs [7,8,9,10]. Copper- and Cu-based NPs are applied in catalysis, in medicine as anticancer and antimicrobial agents, and in agriculture as pesticides [11,12,13].

The intensive application of NPs results in their release into the environment with wastewater as well as emissions into the atmosphere [14]. It has been suggested that wastewater treatment plants have become the main source of NPs in soil and water because the current treatment technologies cannot effectively remove them [15]. Currently, data on the emissions and concentrations of NPs in the environment are limited due to the lack of techniques for their quantitative measurement [16,17]. There is a lack of data about NP concentrations in wastewater; however, several studies reported their concentrations and size in sewage sludge samples. Thus, Ti-, Fe-, Zn-, Sn-, and Pb-containing NPs in the concentration range of 10^7^–10^11^ particles/g were observed in sewage sludge samples from Shanghai. The size of the particles ranged from 10 to 300 nm [16]. The AuNPs with a size of 16 nm and concentration of 1.38 × 10^9^ particles/kg were observed in sewage sludge [18]. 

The use of wastewater for crop irrigation is practiced in many countries around the world [19]. Thus, the use of wastewater containing NPs for irrigation may result in the accumulation of nanosized particles in soil, which in turn can affect soil microbiota and plants. It was reported that ~0.3–1.3 µg/kg and 89.2 µg/kg of TiO_2_ NPs reached soil and sludge-treated soil, respectively, in Europe every year [20]. According to [21], approximately 95% of the nano-sized copper released into the environment will end up in soil and aquatic sediment, where their accumulation may reach concentrations between 50 and 500 μg/L. 

The physical, chemical, and toxicological activity of NPs toward plants and the peculiarities of their absorption by plants all depend on many factors, including the physical and chemical properties of the nanomaterials, type of NPs, type of coating, plant species, and environmental conditions [17,18,22,23].

Among plants subjected to the impact of NPs, special attention is deserved for plants with beneficial properties used for food and medicinal purposes. Among such plants, one that has to be highlighted is curly parsley (*Petroselinum crispum*), a commercially important culinary herb known for its medicinal properties and essential oil [24]. The leaves and roots of parsley are important components of medicinal preparations, while the essential oil of parsley has become broadly popular because of its medicinal, cosmetic, condiment, and preservative properties [24,25]. Parsley segments are used for the treatment of gastrointestinal tract, kidneys, and urinary tract diseases, aid the treatment of diabetes, reduce internal inflammatory processes, and promote liver cleansing. Additionally, *Petroselinum crispum* is used as a carminative, emmenagogic, abortifacient, and nutritive agent [26]. *Petroselinum crispum* has health-promoting properties with the potential to prevent oxidative-stress-related diseases. In addition, parsley is actively used as a condiment, either fresh or dried. Parsley is rich in antioxidants (vitamin C, flavonoid luteolin, vitamin A, and folic acid), and its roots and leaves contain essential oils [27,28].

In a Dehkourdi et al. work [29], the influence of anatase nanoparticles (TiO_2_) in a concentration range of 10–40 mg/mL on the germination of parsley seeds in vitro was studied. The positive effect of TiO_2_ on the percentage of germination, the index of germination, the length of roots and shoots, raw mass, the index of growth strength, and the content of chlorophyll in seedlings was shown. At a concentration of 30 mg/mL, TiO_2_ produced the greatest positive effect, leading to an increase in the level of chlorophyll *a* and *b* compared to control. Besides TiO_2_, in several studies, the beneficial effect of CuNPs on germination and productivity, as well as the fight against plant diseases, has been revealed [22,23]. Experiments with a pre-sowing treatment of barley seeds with CuNPs sized 40–60 nm showed an increase in grain yield by 17.3% compared to control [30]. At the same time, there are studies demonstrating the negative influence of high concentrations of CuNPs on plants, expressed in the disturbance of plants’ metabolism and decline in crop yields [31,32,33]. The investigation of the impact of 10, 30, and 50 nm diameter AuNPs in hydroponic conditions on wheat and tobacco showed that AuNPs with a wide range of sizes and different surface chemistries are bioavailable to plants [34]. It should be mentioned that the uptake of NPs by plants in the soil environment may differ and, as previously mentioned, it depends on many factors.

It is suggested that the uptake of NPs in plants is dependent on NPs’ size and concentration and that it may cause adverse effects. Thus, the main goal of the present study was to assess (i) the uptake of CuNPs and AuNPs introduced in the soils at the concentration range 1–200 mg/L in segments of *Petroselinum crispum*; (ii) changes in the content of chlorophyll and carotenoids in *Petroselinum crispum* exposed to NPs; and (iii) changes in the antioxidant activity of the plant extracts. The obtained results are of great importance for food safety.

## 2. Materials and Methods

### 2.1. Materials

Copper and gold polyvinyl pyrrolidone (PVP)-coated nanoparticles were obtained from the M9 company (Tolyatti, Russia). According to the manufacturer, the nanoparticle solution is stable for two months. The initial concentration of nanoparticles in the solution was 200 mg/L. According to a Rastogi et al. review [35], the NPs may have both a positive and a negative impact on plants, depending on their concentration. Thus, the application of ZnO-NPs in concentrations 10, 50, 100, or 200 ppm increased the length, fresh mass, and dry mass of root and shoot, as well as the leaf area, in tomato plants [36]. To ensure that the data obtained in the present study were comparable with literature values, the effects of the same concentrations of CuNPs and AuNPs on the *Petroselinum crispum* were investigated. In addition, the effects of low concentrations (1 and 5 mg/L), which are far more likely to be present in wastewater, were studied [37]. For the experiments, initial NP solutions were diluted with distillate water to obtain solutions with the following concentrations: 1, 5, 10, 50, and 100 mg/L.

Because experiments were performed in an off-season period, the soil of the brand Terra Vita (Nevatorf Company, Moscow, Russia) was chosen, as it is commonly used for vegetable cultivation in greenhouses. This soil contains high-moor peat of various degrees of decomposition, bio-humus, alluvial sand, agro-perlite, limestone flour, P, K, Ca, Mg, Fe, and microelements [38]. The pH of the soil is 6–6.5. Seeds of curly parsley were purchased from the agricultural company Poisk (Moscow Region, Russia).

### 2.2. Experiment

The effect of NPs was studied on mature, strong plants of *Petroselinum crispum* cultivated in 500 mL pots (one plant in each pot). Plant watering with 10 mL of NP solutions was performed once every 2 days. The control plants were watered with deionized water according to the same experimental scheme. The entire duration of the experiment was 10 days. The temperature and relative humidity of the air in the laboratory were measured daily. Temperature varied between 20 and 21 °C, and humidity varied between 20 and 25%. At the end of the experiment, soil and plant material (roots and leaves) from each pot were collected for analysis. The roots were abundantly washed with distilled water to exclude soil particles. For elemental analysis, samples were dried at 50 °C for 72 h, homogenized, and dried at 105 °C for 24 h. For biochemical analysis, leaves were frozen and stored at a temperature of −80 °C.

### 2.3. Analytical Techniques

The morphology of the NPs was assessed using a transmission electron microscope (TEM), Thermo Scientific Talos F200i (Waltham, MA, USA). TEM images were recorded from the drop-cast film of nanoparticles dissolved in water.

To determine copper and gold content in plant segments, 0.1 g of sample was placed in a Teflon vessel and treated with 3 mL of HNO_3_ (Honeywell Fluka 69%, Sigma-Aldrich, Darmstadt, Germany) and 1 mL of H_2_O_2_ (Sigma-Aldrich 30%, Darmstadt, Germany). Sample digestion was performed in MARS 6 microwave digestion system (CEM, Scottsdale, AZ, USA). Digestion was performed in 1 stage: ramp: temperature 180 °C, time 15 min, power 290–1800 W, and pressure 20 bar. Digests were quantitatively transferred to 10 mL flasks and made up to the volume with bi-distilled water. For soils, 0.2 g of a sample was mixed with 5 mL of HNO_3_, 2 mL of H_2_O_2_, and 1 mL of HF. Sample digestion was performed under the same conditions, and the obtained digest was transferred to 50-mL flasks made up to the volume with bi-distilled water.

The copper content in samples was determined using a high-resolution optical emission spectrometer with inductively coupled plasma, PlasmaQuant PQ 9000 Elite (Analytik Jena, Jena, Germany). The gold content in samples was determined using ICP-MS XSeries II (Thermo Scientific, Waltham, MA, USA). The quality control of the measurements was ensured by analysis of the certified reference materials INCT OBTL-5 (Oriental Basma tobacco leaves) and NIST 2709a (San Joaquin Soil Baseline Trace Element Concentrations). The difference between measured and certified values was less than 5%. 

### 2.4. Biochemical Analysis and Antioxidant Activity

Chlorophyll from fresh parsley biomass was extracted using 90% acetone. The absorbance of the extract was measured at three different wavelengths (645 nm, 652 nm, and 663 nm) using a spectrophotometer. The resulting measurements were used to calculate the total chlorophyll content, in milligrams per 100 g of biomass.

To determine carotene content, extract from fresh parsley biomass was obtained using 96% ethanol and measured by determining the absorbance at 450 nm. The results were calculated in mg/100 g of biomass.

The antioxidant activity of the parsley extract was determined by measuring the reduction in the radical 2,2′-azino-bis (3-ethylbenz-thiazoline-6-sulfonic acid (ABTS) over a period of 6 min. The extract was prepared using a 50% hydro-ethanolic solution. The antioxidant activity was expressed as a percentage of ABTS inhibition.

### 2.5. Data Evaluation

To evaluate nanoparticle uptake, translocation coefficients (*TF*) and bioconcentration factors (*BCF*) were calculated [27,28]:

The translocation coefficient, used to assess metal uptake from the aerial part, was calculated using Equation (1):

(1) $$TF=\frac{C_{L}}{C_{R}}$$

The bioconcentration factor (*BCF*) is defined as the ratio between the content of elements in plants segments and the content in the soil (2):

(2) $$BCF=\frac{C_{R\;or\;L}}{C_{S}}$$

where *C_R_* is the concentration of an element in roots, *C_L_* is the concentration of the element in leaves, and *C_S_* is the concentration of the element in soil.

### 2.6. Statistical Analysis

All experiments and measurements were performed in three repetitions. Statistical analysis was performed by one-way analysis of variance (ANOVA) using Statistica 12 (Student’s *t*-tests). 

## 3. Results

**Figure 1:**
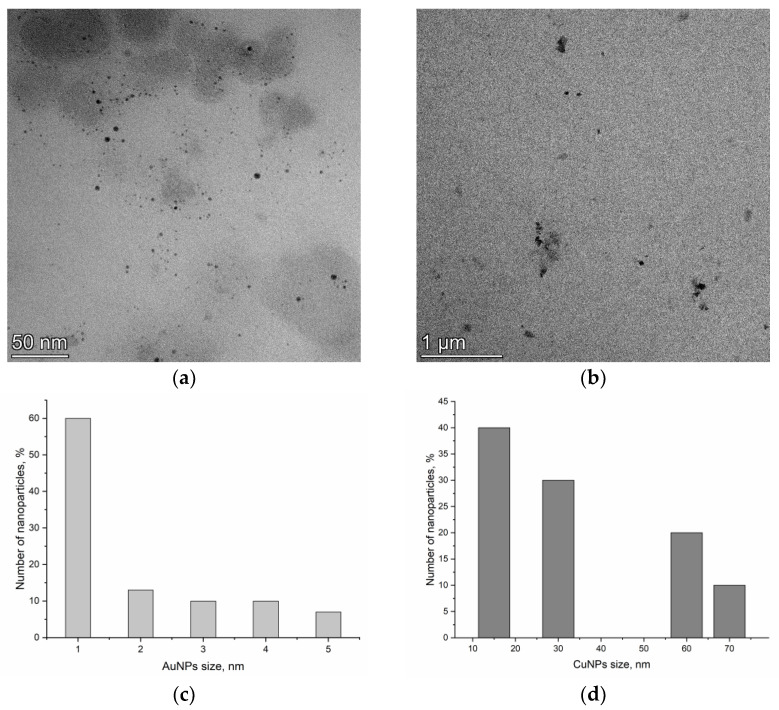
TEM images of (**a**) gold and (**b**) copper nanoparticles and distribution of NP size: (**c**) AuNPs, (**d**) CuNPs.

TEM images of AuNPs and CuNPs, as well as the distribution of NP size, are shown in Figure 1. Both types of NPs had spherical forms. The size of AuNPs ranged from 1 to 5 nm and of CuNPs from 15 to 70 nm.

### 3.1. Gold and Copper Uptake in Parsley Segments

The content of copper in control soil, roots, and leaves was 4.4, 9.65, and 10.4 mg/kg, respectively. Gold content constituted 0.07 mg/kg in roots, 0.16 mg/kg in leaves, and 0.046 mg/kg in soil.

**Figure 2:**
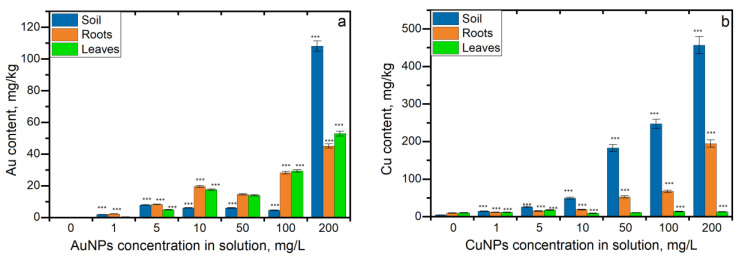
The content of copper and gold in parsley segments grown on soil watered with (**a**) AuNPs and (**b**) CuNPs determined by ICP-MS/OES techniques (NP concentrations 1–200 mg/L, duration of experiment 10 days, *** *p* < 0.0005 for differences between control and samples; *n* = 3, the error bar expresses the standard deviation).

According to data presented in Figure 2a, gold was accumulated in roots and leaves, with a higher concentration remaining in the soil. It should be noted that gold uptake in soil and parsley segments at all applied concentrations was statistically significant (*p* < 0.005) in comparison with the control. The content of gold in the soils at an AuNP concentration of 1 mg/L was 40 times higher than in the control. At AuNP concentrations in the range of 5–100 mg/L, the difference was 100–170 times (Pearson correlation coefficient (r) value being 0.9). At an AuNP concentration of 200 mg/L, its content in soil was 2351 times higher than in control. Accumulation of gold in roots and leaves increased in direct proportion with the AuNP concentration in solution (r for roots 0.9, and for leaves, 0.92). Thus, at an AuNP concentration of 1 mg/L, gold content in roots and leaves was 33 and 2.8 times higher than in control, respectively, while at the highest concentration of 200 mg/L, it was 666 and 326 times higher, respectively. When plants were watered with AuNPs in concentrations of 1–10 mg/L, the content of gold in the roots was higher than in the leaves. The most pronounced difference was observed at an AuNP concentration of 1 mg/L, at which the content of gold in roots was 5 times higher than in leaves. At AuNP concentrations of 5 and 10 mg/L, the content of gold in roots was 1.6 and 1.4 times higher than in leaves, respectively. However, at higher NP concentrations, gold accumulation in leaves exceeded the uptake in roots.

Plant watering with CuNPs resulted in a significant increase in the copper content in the soil. Thus, at the lowest CuNP concentration of 1 mg/L, copper content in biomass increased 3.3 times compared to control, and at the highest concentration of 200 mg/L, it increased 104 times (r = 0.98) (Figure 2b). Copper uptake in roots was directly proportional to the increase in CuNP concentration (r = 0.96). At a CuNP concentration of 1 mg/L, copper content in roots exceeded the control values by 20%. At concentrations 5 and 10 mg/L, the content of copper in roots was 1.6 and 1.9 times higher than control, respectively. At concentrations of 50 and 100 mg/L, increases in copper content by 5 and 7 times, respectively, compared to control were observed. The highest accumulation of copper in roots was noted at a CuNP concentration of 200 mg/L, and it exceeded 20 times its content in control samples. Even the accumulation of copper in leaves was not dependent on CuNP concentration in solution (r = 0.01). It was statistically significant, except at a concentration of 50 mg/L.

### 3.2. Translocation Coefficient (TF) and the Bioconcentration Factor (BCF)

The values of the translocation coefficients (*TF*) and the bioconcentration factors (*BCF*) are presented in Table 1. *BCF* values for CuNPs were less than 1, while *TF* values exceed 1.0 only at NP concentrations of 1 and 5 mg/L. In the case of AuNPs, the uptake of gold from the soil in roots increased with the increase in NP concentrations in the solution, with *BCF* values ranging from 1.4 to 6.8, while at a concentration of 200 mg/L, the values significantly decreased. The same pattern was noticed in the case of NP uptake from the soil in leaves. No uptake of AuNPs from leaves in roots was observed at concentrations 1–50 mg/L, *TF* being lower than 1.0. At concentrations of 100 and 200 mg/L, gold accumulation in leaves from roots was confirmed by *TF* values greater than 1.

### 3.3. Effects of AuNPs and CuNPs on Parsley’s Biochemical Parameters and Antioxidant Activity 

**Figure 3:**
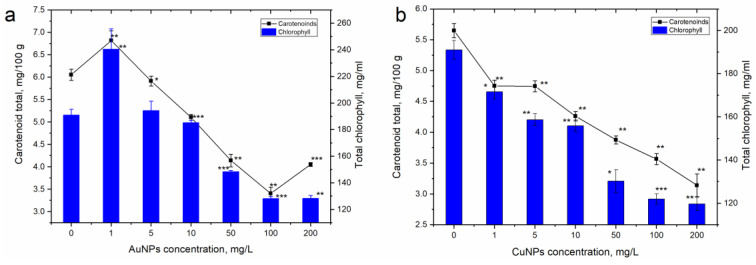
Change in the content of carotenoids and total chlorophyll in the aerial parts of parsley grown on soil watered with (**a**) AuNPs and (**b**) CuNPs (* *p* < 0.05; ** *p* < 0.005; *** *p* < 0.0005 for differences between control and samples; *n* = 3, the error bar expresses the standard deviation).

Figure 3 illustrates the changes in the amounts of carotenoids and total chlorophylls in the extracts obtained from the aerial parts of parsley grown on soil supplemented with different concentrations of AuNPs and CuNPs.

The obtained results demonstrate different patterns of responses of the two types of NPs. Thus, in the case of AuNPs at low concentrations (1 and 5 mg/L), the content of pigments in the plants increased or remained on a level characteristic of the control. The AuNPs introduced in the soil in a concentration of 1 mg/L led to a significant increase in the quantity of carotenoid pigments (by 12.7%) and total chlorophyll (by 25.9%) compared to the control. The concentration of 5 mg/L did not affect the amount of chlorophyll and insignificantly reduced the carotenoid content. Soil watering with AuNPs in concentrations higher than 10 mg/L caused an important decrease in the quantity of carotenoids by 15.7−43.8% compared to the control. The total chlorophyll content decreased by 22.3−32.9% compared to the control, starting with the AuNP concentration of 50 mg/L. 

CuNPs, even at the lowest applied concentration, caused a significant decrease in both carotenoid and total chlorophyll content (by 10.2–37.4% compared to the control). Soil watering with CuNPs in concentrations of 1 and 5 mg/L resulted in a rather similar reduction in carotenoid amount: by 15.9% (*p* < 0.05) and by 16.0 (*p* < 0.005), respectively. The increase in CuNP concentrations in the soil led to the continuous decrease in the carotenoid content by 24.5–44.4% compared to the control. 

**Figure 4:**
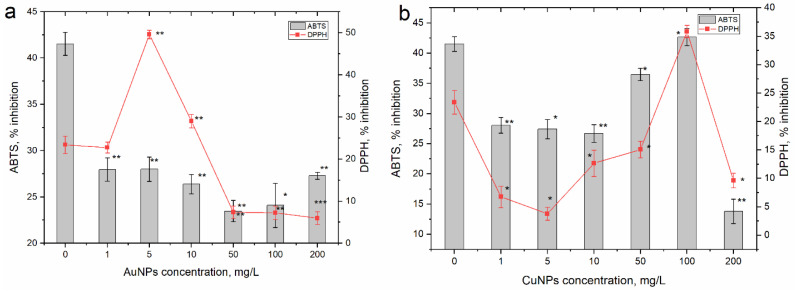
Modification of the antioxidant activity of extracts obtained from the aerial parts of parsley grown on soil watered with (**a**) gold and (**b**) copper nanoparticles (* *p* < 0.05; ** *p* < 0.005; *** *p* < 0.0005 for differences between control and samples; *n* = 3, the error bar expresses the standard deviation).

The changes in the antioxidant activity of extracts obtained from the parsley grown on soil watered with AuNPs and CuNPs were more complex and complicated to describe (Figure 4).

In the case of AuNPs, the values of the ABTS test were low but rather homogeneous in all experimental variants. Compared to the control, extracts obtained from parsley grown on soil watered with AuNPs in the concentration range of 1–200 mg/L lost their ability to reduce the radical cation ABTS by 32.7 to 43.5%. While soil watering with AuNPs at the concentration of 1 mg/L, the capacity of the extract to inhibit the DPPH radical in the sample was at the level of the control; however, at an AuNP concentration of 5 mg/L, the antioxidant capacity of the extract increased by 2 times compared to the control and exhibited 49.6% inhibition. At an NP concentration of 10 mg/L, the extract was also more active than the control, and it decreased by 24% compared to the control. Three concentrations resulted in a very significant decrease in DPPH radical-reducing activity. Thus, the activity of the extracts from the plants watered with AuNPs in concentrations of 50, 100, and 200 mg/L was 68.4–74.6% lower compared to the control.

Important fluctuations in antioxidant activity also occurred in extracts obtained from plants grown on soil watered with CuNPs. In these cases, a reduction in the capacity to reduce the DPPH radical and the ABTS cation radical, except at the concentration of 100 mg/L, was observed. At CuNP concentrations of 1, 5, and 10 mg/L, the values of the ABTS test decreased compared to the control by 32.4–35.7%. At the concentration of 50 mg/L, this discrepancy was significantly reduced and the difference compared to the control reached 12.1%; at the concentration of 100 mg/L, the activity of the extract from the experimental variant was comparable to the control. Doubling of the CuNP concentration led to a drastic decrease—by three times—in the antioxidant activity. The same pattern of modification of the antioxidant activity was observed in the case of the DPPH test. At CuNP concentrations of 1–200 mg/L, the antiradical activity of the extracts from the experimental plants was 35.5–83.7% lower compared to the control. In the case of this test, in the variant with a CuNP concentration of 100 mg/L, the ability to reduce the DPPH radical increased by 1.5 times compared to the control.

## 4. Discussion

Exposure to NPs leads to their eventual accumulation in the soil ecosystem, absorption by edible plants, and uptake by the human body through the food chain [15]. 

Plant watering with AuNPs in the concentration range of 1–200 mg/L did not cause visible deterioration of parsley. However, in parsley plants watered with solutions of CuNPs at concentrations 50, 100, and 200 mg/L, wilting of the leaves on the 7th day of the experiment was observed. 

The accumulation of gold in soils and uptake by plants was directly proportional to AuNP concentration in the solution. The maximum accumulation of gold was attained at the highest NP concertation of 200 mg/L. At low AuNP concentrations (up to 10 mg/L), gold predominantly accumulated in roots, while at higher NP concentrations, its accumulation in leaves exceeded the uptake in roots. The AuNPs’ active uptake in parsley segments can be explained by their small size [39,40]. It was shown previously that AuNPs with a diameter of 3.5 nm entered plants easily through the root system, while there was no accumulation of NPs with a size of 18 nm [41]. Thus, it can be suggested that in the presented study, AuNPs were accumulated as single particles and no agglomeration occurred. AuNPs are considered relatively stable in the environment, and nanoparticles were the dominant form of gold accumulated in different parts of carrot, radish, lettuce, and potato [42]. Further research utilizing a transmission electron microscope could provide information about the aggregation of nanoparticles in plants.

The obtained results correlate well with other published works, which show that AuNPs accumulate in the root systems of plants and are translocated to the aerial parts. Thus, Malejko et al. [42] studied AuNP uptake in carrot, radish, lettuce, and potato cultivated to maturity. AuNPs were accumulated differently in the studied plants. In radish, gold accumulation was higher in leaves, while its uptake in edible potato parts was negligible. AuNPs of the size 3.5 nm were accumulated in the stems, leaves, and roots of tobacco in the metallic form [41]. Judy et al. [34] studied the effect of 10, 30, and 50 nm diameter AuNPs coated with tannate or citrate on *Nicotiana tabacum* L. cv Xanthi and *Triticum aestivum* and showed accumulation of both types of NPs in tobacco but no accumulation in wheat. No significant transport of AuNPs from the roots into the shoots of *Hordeum vulgare* L. occurred within the 2-week exposure period [43].

The content of copper in the soil increased with the increase in NP concentration in the solution. The relatively low uptake of copper in roots at CuNP concentrations in the range of 1–100 mg/L was replaced by a considerable increase at a concentration of 200 mg/L. Low uptake of CuNPs at concentrations in the range of 1–100 mg/L can be associated with the NP agglomeration reducing the actual concentration of NPs available for plant uptake [44]. High copper uptake at a CuNP concentration of 200 mg/L can be explained by the presence of NP agglomerates that are attached to the surface of the roots [43]. The uptake of copper in leaves was negligible.

The obtained results are in agreement with a Tamez et al. study [45] that showed that CuNPs were mainly accumulated in roots of sugarcane and the translocation of copper to aerial tissues was minimal. Studying the accumulation of CuNP in *Cucumis sativus*, the authors showed higher copper accumulation in the roots compared to the shoots and associated it with the NPs’ toxicity, which was manifested by the decrease in the level of photosynthetic pigments and plant biomass [46]. A low rate of CuNP translocation from roots in aerial tissues can be associated with the activation of the root defense mechanism against nano-Cu stress [47]. 

NP uptake plant by plant is affected by multiple factors such as particle size, surface functionalization, morphology, exposure conditions, plant species, plant growth stage, root integrity (damage or disease), and rhizosphere processes [26,35,42,48].

The values of *BCF* calculated for leaves and roots of parsley watered with CuNPs indicated a rather weak transfer of copper from soil to plant segments. The translocation of copper from the roots to the aerial parts decreased with an increase in the CuNP concentration in solutions. The distribution of copper content in the soil–plant system can be represented as follows: soil > root > leaves. CuNPs can release metal ions, causing cellular oxidative stress and poisoning plants. It is known that CuNPs can alter the activity of antioxidant enzymes and inhibit the accumulation of nutrients [49]. Wilting of the leaves, as well as the results of the biochemical tests, confirm the toxicity of CuNPs.

In the case of AuNPs, the calculated *BCF* and *TF* coefficients indicate the influx of gold into the aerial parts of plants through the root system. The highest *BCF* values for roots and leaves were obtained at AuNP concentrations of 10, 50, and 100 mg/L. At the highest AuNP concentration of 200 mg/L, even the gold content in the soil increased significantly, and a sharp decrease in the ability of parsley to accumulate gold in plant segments was observed, which can be associated with the toxicity of high concentrations of AuNPs.

The growth of parsley on soil watered with AuNPs and CuNPs led to changes in the pigment content and the antioxidant activity of extracts obtained from the biomass of the aerial parts of the plants. The obtained results demonstrated different patterns of responses to the two types of NPs. Thus, at low concentrations, AuNPs had a positive effect on the quantity of pigments. At concentrations higher than 10 mg/L, a decrease in the pigment content was noted. It is suggested that high concentrations of AuNPs have a toxic effect on parsley, and the increase in pigment quantity at the lowest concentration applied is most likely an expression of hormesis. CuNPs, at all applied concentrations, led to a decrease in pigment content. Although the decreasing trend in the content of these pigments in parsley suggests a dose-dependent effect, an inversely proportional relationship was not attained, and the rate of decrease dropped with the increase in the nanoparticle concentrations. 

Antioxidant activity was measured using two methods: one based on electron transfer (ABTS test) and the other on proton transfer (DPPH test). The observed responses were different for the two types of NPs. Thus, regarding AuNPs, the values of the ABTS test were low but rather homogeneous in all experimental variants. Compared to the control, in the extracts obtained from parsley grown on soil watered with AuNPs in the concentration range of 1–200 mg/L, the ability to reduce the ABTS cation radical was lost. Thus, in all the experimental variants, a state of stress caused by the presence of AuNPs in the soil was observed. The results of the DPPH test differ from those of the ABTS test, but they also reflect a state of stress in parsley.

Important fluctuations in antioxidant activity also occurred in extracts obtained from plants grown on soil watered with CuNPs. In these variants, a reduction in the capacity to reduce the DPPH radical and the ABTS cation radical, except at the concentration of 100 mg/L, was observed. 

Such a complicated dependence between the concentration of NPs used for watering and the obtained response reaction may be caused by the aggregation ability of NPs; however, this needs to be studied separately, and it was not an objective of the present study. It is known that the effects of different NPs on plant systems are determined by several factors, such as the chemical nature of the particles, their size, and reactivity, but the most important is the nanoparticle concentration, which comes into contact with the plant [36,37]. Depending on the combinations of the mentioned factors but also on the plant species, the effects of nanoparticles can be either inhibitory or stimulating [50].

The inhibitory effects were observed at high concentrations of CuNPs. For example, the growth of *Phaseolus radiates* shoots was not affected by low concentrations of CuNPs, while the negative effects appeared starting from the concentration of 800 mg/L [51]. In other species, these negative effects can occur at much lower concentrations. The toxicity of CuNPs in *Triticum aestivum* was manifested starting from NP concentrations of 200 mg/L [52]. Some authors have reported changes in the photosynthetic apparatus of plants upon the action of CuNPs or copper oxide. For example, in *Lemna gibba*, copper oxide nanoparticles applied in doses up to 4 g/L produced a dose-dependent decrease in plant growth by decreasing PSII activity and inactivating the reaction centers of this photosynthetic system [53].

A decrease in photosystem II yield under the action of copper oxide nanoparticles was attested in *Oryza sativa*. At NP concentrations between 2.5 and 1000 mg/L, there was a dose-dependent decrease in the number of thylakoids per grain, photosynthetic rate, transpiration rate, and stomatal conductance. The authors also reported a reduction in the content of photosynthetic pigments in plants, but also an increased level of oxidative and osmotic stress and a higher level of expression of the antioxidant enzymes APX and SOD [54]. Low concentrations of CuNPs were able to stimulate the bio-synthesis process in *Elodea densa*, but the stimulating effects were noticeable at concentrations up to 0.25 mg/L [55].

In the case of AuNPs, it is also known that depending on the NP concentration and the plant species, positive results can be obtained, expressed in the increase in the seeds’ germination capacity. The stimulating effect of AuNPs was reported for *Lactuca sativa*, *Cucumis sativus*, *Brassica juncea*, etc. [42,56]. The negative effects of NPs on plants usually are observed under the condition of application in high doses. One of the possible mechanisms of nanoparticle toxicity is the suppression of functions of the AQP proteins involved in the transfer of substances through cell membranes. Furthermore, high nanoparticle concentrations cause structural damage in cells, which ultimately leads to the dysregulation of vital metabolic processes [51]. At the same time, the positive effects caused by nanoparticles are attributed to the hormesis effect or sophisticated mechanisms of gene transport and transformation under the influence or with the participation of nanoparticles, for example, gold ones [57].

## 5. Conclusions

The uptake of AuNPs and CuNPs in parsley and their impact on the plant’s biochemical composition and antiradical activity were investigated in a ten-day experiment. Despite the fact that NPs were used in the same range of concentrations, 1–200 mg/L, their accumulation in soil and translocation in roots and leaves was different. Thus, with the application of CuNPs, copper content in soil increased 3.3–104 times compared to control, and in roots, it increased 1.6–20 times. Copper uptake in leaves was not affected by the NP concentrations. In contrast, AuNPs were actively accumulated in both roots and the aerial parts. At AuNP concentrations of 1–10 mg/L, gold uptake changed in the order soil > roots > leaves, while at concentration range 50–200 mg/L, the order was soil > leaves > roots. Soil samples accumulated 40–2351 times more gold in comparison with the control. In roots and leaves, maximum gold accumulation of 45 and 53 mg/kg, respectively, was attained at NP concentrations of 200 mg/L.

AuNPs and CuNPs generated stress conditions in parsley, expressed by the reduction in the content of pigments and change in the antioxidant capacity. The magnitude of the generated effects is determined by both the type of nanoparticles and their concentration. The observed stimulating effects are most likely an expression of hormesis.

## Figures and Tables

**Table 1:** Translocation coefficients (*TF*) and the bioconcentration factors (*BCF*) in parsley segments grown on soil watered with AuNPs and CuNPs.

Concentration of NPs in Solution, mg/L	*BCF*		*TF*
	Roots	Leaves	
CuNPs			
1	0.78	0.8	1.03
5	0.57	0.67	1.17
10	0.38	0.18	0.48
50	0.29	0.06	0.2
100	0.27	0.06	0.2
200	0.43	0.03	0.06
AuNPs			
1	1.2	0.24	0.2
5	1.04	0.62	0.6
10	3.21	2.88	0.9
50	2.41	2.31	0.96
100	6.08	6.32	1.04
200	0.42	0.49	1.17
